# Crystal structure of (1-eth­oxy­ethyl­idene)di­methyl­aza­nium tetra­phenyl­borate

**DOI:** 10.1107/S2056989015022252

**Published:** 2015-11-25

**Authors:** Ioannis Tiritiris, Stefan Saur, Willi Kantlehner

**Affiliations:** a Fakultät Chemie/Organische Chemie, Hochschule Aalen, Beethovenstrasse 1, D-73430 Aalen, Germany

**Keywords:** crystal structure, (eth­oxy­ethyl­idene)di­methyl­aza­nium, tetra­phenyl­borate, salt, C—H⋯π inter­actions

## Abstract

In the cation of the title salt, C_6_H_14_NO^+^·C_24_H_20_B^−^, the C—N bond lengths are 1.297 (2), 1.464 (2) and 1.468 (2) Å, indicating double- and single-bond character, respectively. The C—O bond length of 1.309 (2) Å shows double-bond character, pointing towards charge delocalization within the NCO plane of the iminium ion. In the crystal, C—H⋯π inter­actions between the iminium H atoms and the phenyl C atoms of the anion are present. The phenyl rings form aromatic pockets, in which the iminium ions are embedded.

## Related literature   

For acetalization reactions with carboxamide-dialkyl sulfate adducts, see: Kantlehner *et al.* (1980 ▸). For the crystal structure of (meth­oxy­methyl­idene)di­methyl­aza­nium tetra­phenyl­borate aceto­nitrile monosolvate, see: Tiritiris *et al.* (2014*a* ▸). For the crystal structure of (but­oxy­methyl­idene)di­methyl­aza­nium tetra­phenyl­borate aceto­nitrile monosolvate, see: Tiritiris *et al.* (2014*b* ▸). For the crystal structure of (eth­oxy­ethyl­idene)di­methyl­aza­nium ethyl sulfate, see: Tiritiris *et al.* (2015 ▸). For the crystal structure analysis of alkali metal tetra­phenyl­borates, see: Behrens *et al.* (2012 ▸). For the use of intensity quotients and differences in absolute structure refinement, see: Parsons *et al.* (2013 ▸).
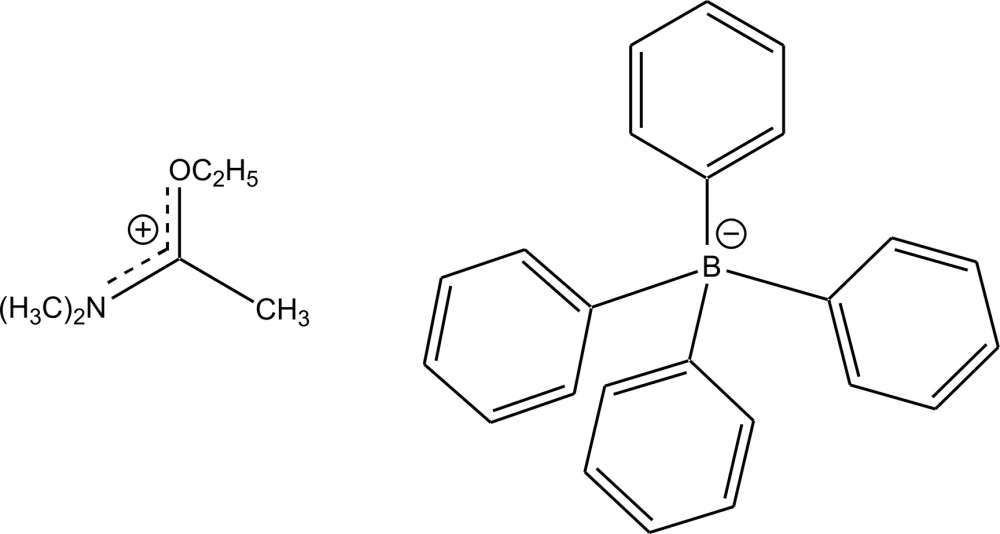


## Experimental   

### Crystal data  

C_6_H_14_NO^+^·C_24_H_20_B^−^
*M* *_r_* = 435.39
Orthorhombic, 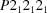

*a* = 9.9849 (6) Å
*b* = 11.5293 (7) Å
*c* = 21.1980 (12) Å
*V* = 2440.3 (3) Å^3^
*Z* = 4
Mo *K*α radiation
μ = 0.07 mm^−1^
*T* = 100 K
0.54 × 0.39 × 0.18 mm

### Data collection  

Bruker Kappa APEXII DUO diffractometer
Absorption correction: multi-scan (Blessing, 1995 ▸) *T*_min_ = 0.726, *T*_max_ = 0.746
33864 measured reflections
7520 independent reflections
6825 reflections with *I* > 2σ(*I*)
*R*_int_ = 0.029

### Refinement  

*R*[*F* ^2^ > 2σ(*F* ^2^)] = 0.037
*wR*(*F* ^2^) = 0.095
*S* = 1.03
7520 reflections
302 parameters
H-atom parameters constrained
Δρ_max_ = 0.25 e Å^−3^
Δρ_min_ = −0.23 e Å^−3^

### 

Data collection: *APEX2* (Bruker, 2008 ▸); cell refinement: *SAINT* (Bruker, 2008 ▸); data reduction: *SAINT*; program(s) used to solve structure: *SHELXS97* (Sheldrick, 2008 ▸); program(s) used to refine structure: *SHELXL2014* (Sheldrick, 2015 ▸); molecular graphics: *DIAMOND* (Brandenburg & Putz, 2005 ▸); software used to prepare material for publication: *SHELXL2014*.

## Supplementary Material

Crystal structure: contains datablock(s) I, global. DOI: 10.1107/S2056989015022252/zl2652sup1.cif

Structure factors: contains datablock(s) I. DOI: 10.1107/S2056989015022252/zl2652Isup2.hkl

Supporting information file. DOI: 10.1107/S2056989015022252/zl2652Isup3.cml

**Fig. 1.**
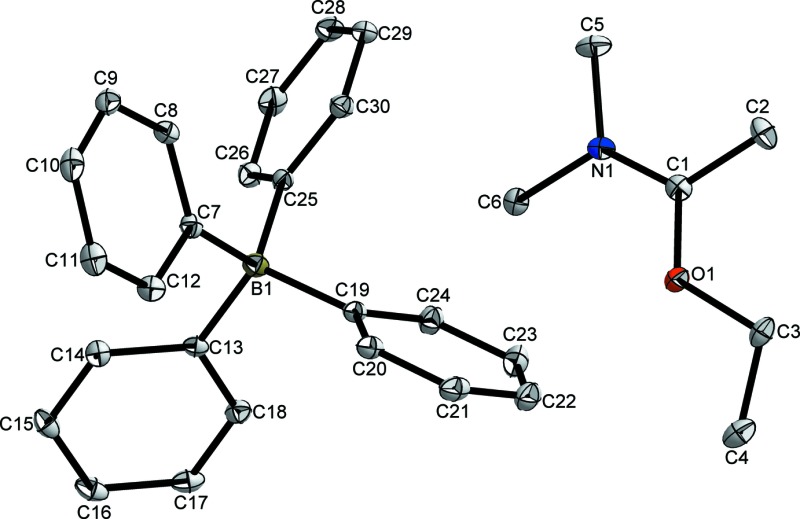
The structure of the title compound with displacement ellipsoids at the 50% probability level. All hydrogen atoms were omitted for the sake of clarity.

. DOI: 10.1107/S2056989015022252/zl2652fig1.tif
The structure of the title compound with displacement ellipsoids at the 50% probability level. All hydrogen atoms were omitted for the sake of clarity.

**Fig. 2.**
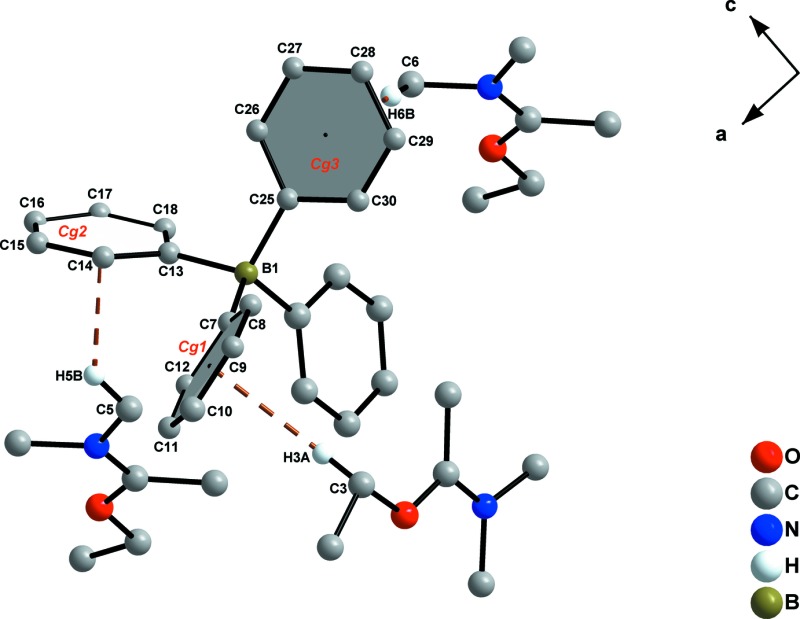
C—H···π interactions (brown dashed lines) between the hydrogen atoms of the guanidinium ion and the phenyl carbon atoms (centroids) of the tetraphenylborate ion.

. DOI: 10.1107/S2056989015022252/zl2652fig2.tif
C—H⋯π inter­actions (brown dashed lines) between the hydrogen atoms of the guanidinium ion and the phenyl carbon atoms (centroids) of the tetra­phenyl­borate ion.

CCDC reference: 1437994

Additional supporting information:  crystallographic information; 3D view; checkCIF report

## Figures and Tables

**Table 1:** Hydrogen-bond geometry (Å, °) *Cg*1, *Cg*2 and *Cg*3 are the centroids of the C7–C12, C13–C18 and C25–C30 rings, respectively.

*D*—H⋯*A*	*D*—H	H⋯*A*	*D*⋯*A*	*D*—H⋯*A*
C3—H3*A*⋯*Cg*1	0.99	2.67	3.572 (2)	151
C5—H5*B*⋯*Cg*2	0.98	2.70	3.450 (2)	134
C6—H6*B*⋯*Cg*3	0.98	2.72	3.692 (2)	175

## supplementary crystallographic information

### S1. Comment

Carboxamide-dialkyl sulfate adducts are salts that can be used for acetalization reactions (Kantlehner *et al.*, 1980). The 1:1 adduct of *N*,*N*-dimethylacetamide and diethyl sulfate, known as (ethoxyethylidene)dimethylazanium ethyl sulfate (Tiritiris *et al.*, 2015) is one of them. By reaction with sodium tetraphenylborate in acetonitrile, it was possible to achieve an anion exchange and to obtain the title compound. The structure analysis reveals that the bond lengths and angles in the cation are in very good agreement with the data obtained from the structure analysis of (ethoxyethylidene)dimethylazanium ethyl sulfate (Tiritiris *et al.*, 2015). In the tetraphenylborate salt, the C5–N1 bond length is 1.468 (2) Å, C6–N1 = 1.464 (2) Å and C1–N1 = 1.297 (2) Å, showing single and double bond character, respectively. The C–N1–C angles are: 115.24 (12)° (C5–N1–C6), 122.11 (13)° (C1–N1–C5) and 122.65 (13)° (C1–N1–C6), which indicates a nearly trigonal-planar surrounding of the nitrogen centre by the carbon atoms (Fig. 1). The C–O bond length shows with 1.309 (2) Å double bond character. The positive charge is completely delocalized on the plane formed by the atoms N1, C1 and O1 (Fig. 1). The C3–O1 bond length of 1.471 (2) Å is indicating single bond character. The bond lengths and angles in the tetraphenylborate ions are in good agreement with the data from the crystal structure analysis of the alkali metal tetraphenylborates (Behrens *et al.*, 2012). C–H···π interactions between the iminium hydrogen atoms of –N(CH_3_)_2_ and –CH_2_ groups and the phenyl carbon atoms (centroids: *Cg*1 = C7—C12, *Cg*2 = C13—C18 and *Cg*3 = C25—C30) of the tetraphenylborate ion are present (Fig. 2), ranging from 2.67 to 2.72 Å (Tab. 1). Such a type of interactions were also observed in the iminium salts (methoxymethylidene)dimethylazanium tetraphenylborate acetonitrile monosolvate (Tiritiris *et al.*, 2014*a*) and (butoxymethylidene)dimethylazanium tetraphenylborate acetonitrile monosolvate (Tiritiris *et al.*, 2014*b*). The phenyl rings form aromatic pockets, in which the guanidinium ions are embedded.

### S2. Experimental

The title compound was obtained by anion exchange reaction. 1.00 g (3.66 mmol) of (ethoxyethylidene)dimethylazanium ethyl sulfate (Tiritiris *et al.*, 2015) was dissolved in 20 ml acetonitrile and 1.25 g (3.66 mmol) of sodium tetraphenylborate in 20 ml acetonitrile was added. After stirring for one hour at room temperature, the precipitated sodium ethyl sulfate was filtered off. The title compound crystallized from a saturated acetonitrile solution after several hours at 273 K, forming colorless single crystals. Yield: 1.35 g (85%).

### S3. Refinement

The title compound crystallizes in the non-centrosymmetric space group *P2_1_2_1_2_1_*; however, in the absence of significant anomalous scattering effects, the determined Flack parameter *x* = -0.2 (4) (Parsons *et al.*, 2013) is essentially meaningless. The hydrogen atoms of the methyl groups were allowed to rotate with a fixed angle around the C–N and C–C bonds to best fit the experimental electron density, with *U*_iso_(H) set to 1.5 *U*_eq_(C) and d(C—H) = 0.98 Å. The remaining H atoms were placed in calculated positions with d(C—H) = 0.99 Å (H atoms in CH_2_ groups) and (C—H) = 0.95 Å (H atoms in aromatic rings). They were refined using a riding model, with *U*_iso_(H) set to 1.2*U*_eq_(C).

### Crystal data

**Table:**

C_6_H_14_NO^+^·C_24_H_20_B^−^	*F*(000) = 936
*M**_r_* = 435.39	*D*_x_ = 1.185 Mg m^−^^3^
Orthorhombic, *P*2_1_2_1_2_1_	Mo *K*α radiation, λ = 0.71073 Å
Hall symbol: P 2ac 2ab	Cell parameters from 6826 reflections
*a* = 9.9849 (6) Å	θ = 2.0–30.6°
*b* = 11.5293 (7) Å	µ = 0.07 mm^−^^1^
*c* = 21.1980 (12) Å	*T* = 100 K
*V* = 2440.3 (3) Å^3^	Block, colorless
*Z* = 4	0.54 × 0.39 × 0.18 mm

### Data collection

**Table:**

Bruker Kappa APEXII DUO diffractometer	7520 independent reflections
Radiation source: fine-focus sealed tube	6825 reflections with *I* > 2σ(*I*)
Triumph monochromator	*R*_int_ = 0.029
φ scans, and ω scans	θ_max_ = 30.6°, θ_min_ = 1.9°
Absorption correction: multi-scan (Blessing, 1995)	*h* = −14→14
*T*_min_ = 0.726, *T*_max_ = 0.746	*k* = −16→16
33864 measured reflections	*l* = −27→30

### Refinement

**Table:**

Refinement on *F*^2^	Primary atom site location: structure-invariant direct methods
Least-squares matrix: full	Secondary atom site location: difference Fourier map
*R*[*F*^2^ > 2σ(*F*^2^)] = 0.037	Hydrogen site location: inferred from neighbouring sites
*wR*(*F*^2^) = 0.095	H-atom parameters constrained
*S* = 1.03	*w* = 1/[σ^2^(*F*_o_^2^) + (0.0527*P*)^2^ + 0.3379*P*] where *P* = (*F*_o_^2^ + 2*F*_c_^2^)/3
7520 reflections	(Δ/σ)_max_ < 0.001
302 parameters	Δρ_max_ = 0.25 e Å^−^^3^
0 restraints	Δρ_min_ = −0.23 e Å^−^^3^

### Special details

**Table:**

Geometry. All e.s.d.'s (except the e.s.d. in the dihedral angle between two l.s. planes) are estimated using the full covariance matrix. The cell e.s.d.'s are taken into account individually in the estimation of e.s.d.'s in distances, angles and torsion angles; correlations between e.s.d.'s in cell parameters are only used when they are defined by crystal symmetry. An approximate (isotropic) treatment of cell e.s.d.'s is used for estimating e.s.d.'s involving l.s. planes.
Refinement. Refinement of *F*^2^ against ALL reflections. The weighted *R*-factor *wR* and goodness of fit *S* are based on *F*^2^, conventional *R*-factors *R* are based on *F*, with *F* set to zero for negative *F*^2^. The threshold expression of *F*^2^ > σ(*F*^2^) is used only for calculating *R*-factors(gt) *etc*. and is not relevant to the choice of reflections for refinement. *R*-factors based on *F*^2^ are statistically about twice as large as those based on *F*, and *R*- factors based on ALL data will be even larger.

### Fractional atomic coordinates and isotropic or equivalent isotropic displacement parameters (Å^2^)

**Table:**

	*x*	*y*	*z*	*U*_iso_*/*U*_eq_	
O1	0.30998 (11)	0.52467 (9)	0.12549 (5)	0.0192 (2)	
C1	0.21195 (15)	0.59672 (13)	0.11353 (7)	0.0165 (3)	
N1	0.21207 (12)	0.69225 (11)	0.14580 (6)	0.0165 (2)	
C2	0.10847 (17)	0.57082 (16)	0.06546 (7)	0.0233 (3)	
H2A	0.0346	0.5281	0.0851	0.035*	
H2B	0.1479	0.5237	0.0318	0.035*	
H2C	0.0745	0.6436	0.0477	0.035*	
C3	0.31599 (17)	0.41096 (13)	0.09413 (7)	0.0210 (3)	
H3A	0.3350	0.4204	0.0486	0.025*	
H3B	0.2299	0.3693	0.0989	0.025*	
C4	0.42626 (19)	0.34548 (14)	0.12555 (9)	0.0269 (3)	
H4A	0.5109	0.3873	0.1200	0.040*	
H4B	0.4337	0.2682	0.1066	0.040*	
H4C	0.4067	0.3379	0.1707	0.040*	
C5	0.10603 (17)	0.77963 (15)	0.13914 (8)	0.0238 (3)	
H5A	0.1223	0.8259	0.1011	0.036*	
H5B	0.1060	0.8305	0.1762	0.036*	
H5C	0.0190	0.7408	0.1357	0.036*	
C6	0.31634 (16)	0.72012 (13)	0.19196 (7)	0.0210 (3)	
H6A	0.2801	0.7118	0.2347	0.031*	
H6B	0.3466	0.8001	0.1856	0.031*	
H6C	0.3922	0.6671	0.1865	0.031*	
B1	0.74360 (16)	0.96058 (13)	0.13424 (7)	0.0134 (3)	
C7	0.81051 (14)	1.07099 (12)	0.09675 (6)	0.0144 (2)	
C8	0.74193 (16)	1.17297 (13)	0.08187 (7)	0.0185 (3)	
H8	0.6496	1.1784	0.0923	0.022*	
C9	0.80315 (18)	1.26719 (13)	0.05239 (7)	0.0224 (3)	
H9	0.7524	1.3349	0.0435	0.027*	
C10	0.93698 (18)	1.26280 (14)	0.03606 (7)	0.0222 (3)	
H10	0.9785	1.3261	0.0151	0.027*	
C11	1.00906 (16)	1.16413 (14)	0.05094 (7)	0.0212 (3)	
H11	1.1016	1.1600	0.0408	0.025*	
C12	0.94734 (15)	1.07112 (13)	0.08062 (7)	0.0175 (3)	
H12	0.9996	1.0047	0.0905	0.021*	
C13	0.83345 (14)	0.93481 (12)	0.19804 (6)	0.0137 (2)	
C14	0.91903 (14)	1.01811 (13)	0.22415 (7)	0.0160 (3)	
H14	0.9228	1.0927	0.2052	0.019*	
C15	0.99888 (15)	0.99624 (15)	0.27663 (7)	0.0198 (3)	
H15	1.0568	1.0550	0.2922	0.024*	
C16	0.99417 (16)	0.88928 (15)	0.30619 (7)	0.0213 (3)	
H16	1.0504	0.8729	0.3412	0.026*	
C17	0.90577 (16)	0.80661 (14)	0.28360 (7)	0.0204 (3)	
H17	0.8984	0.7340	0.3044	0.024*	
C18	0.82782 (15)	0.82896 (13)	0.23073 (7)	0.0168 (3)	
H18	0.7684	0.7704	0.2161	0.020*	
C19	0.74159 (14)	0.84278 (12)	0.09026 (6)	0.0150 (3)	
C20	0.82655 (15)	0.82106 (13)	0.03890 (6)	0.0175 (3)	
H20	0.8890	0.8791	0.0268	0.021*	
C21	0.82289 (18)	0.71751 (14)	0.00481 (7)	0.0220 (3)	
H21	0.8823	0.7065	−0.0297	0.026*	
C22	0.73336 (19)	0.63092 (15)	0.02089 (8)	0.0257 (3)	
H22	0.7312	0.5601	−0.0020	0.031*	
C23	0.64692 (18)	0.64933 (14)	0.07098 (8)	0.0262 (3)	
H23	0.5842	0.5911	0.0825	0.031*	
C24	0.65189 (16)	0.75297 (13)	0.10442 (7)	0.0204 (3)	
H24	0.5915	0.7634	0.1386	0.025*	
C25	0.58849 (14)	0.98927 (12)	0.15445 (6)	0.0140 (2)	
C26	0.54660 (14)	1.00130 (13)	0.21695 (7)	0.0174 (3)	
H26	0.6106	0.9916	0.2497	0.021*	
C27	0.41480 (16)	1.02689 (14)	0.23328 (7)	0.0214 (3)	
H27	0.3906	1.0341	0.2765	0.026*	
C28	0.31913 (15)	1.04184 (13)	0.18703 (8)	0.0210 (3)	
H28	0.2293	1.0601	0.1979	0.025*	
C29	0.35655 (15)	1.02972 (13)	0.12432 (8)	0.0192 (3)	
H29	0.2919	1.0395	0.0919	0.023*	
C30	0.48800 (14)	1.00335 (13)	0.10890 (7)	0.0163 (3)	
H30	0.5109	0.9944	0.0657	0.020*	

### Atomic displacement parameters (Å^2^)

**Table:**

	*U*^11^	*U*^22^	*U*^33^	*U*^12^	*U*^13^	*U*^23^
O1	0.0211 (5)	0.0158 (5)	0.0209 (5)	0.0032 (4)	−0.0016 (4)	−0.0033 (4)
C1	0.0166 (6)	0.0184 (6)	0.0144 (6)	−0.0012 (5)	0.0015 (5)	0.0026 (5)
N1	0.0149 (6)	0.0174 (6)	0.0173 (5)	0.0023 (4)	0.0008 (4)	0.0024 (4)
C2	0.0216 (7)	0.0301 (8)	0.0182 (7)	−0.0033 (6)	−0.0042 (6)	0.0008 (6)
C3	0.0261 (8)	0.0157 (6)	0.0211 (7)	0.0004 (6)	0.0048 (6)	−0.0046 (5)
C4	0.0313 (9)	0.0184 (7)	0.0311 (8)	0.0058 (6)	0.0045 (7)	−0.0023 (6)
C5	0.0200 (7)	0.0230 (7)	0.0285 (8)	0.0090 (6)	0.0034 (6)	0.0038 (6)
C6	0.0218 (7)	0.0180 (6)	0.0232 (7)	−0.0001 (6)	−0.0033 (6)	−0.0022 (5)
B1	0.0127 (6)	0.0143 (6)	0.0132 (6)	0.0006 (5)	0.0005 (5)	−0.0001 (5)
C7	0.0159 (6)	0.0154 (6)	0.0118 (5)	−0.0007 (5)	−0.0007 (5)	0.0001 (5)
C8	0.0172 (6)	0.0178 (6)	0.0207 (7)	0.0007 (5)	−0.0051 (5)	0.0010 (5)
C9	0.0297 (8)	0.0148 (6)	0.0227 (7)	−0.0014 (6)	−0.0102 (6)	0.0031 (5)
C10	0.0315 (8)	0.0199 (7)	0.0153 (6)	−0.0096 (6)	−0.0039 (6)	0.0031 (5)
C11	0.0213 (7)	0.0254 (8)	0.0168 (6)	−0.0063 (6)	0.0026 (6)	0.0008 (6)
C12	0.0173 (6)	0.0190 (7)	0.0162 (6)	0.0009 (5)	0.0018 (5)	0.0019 (5)
C13	0.0121 (6)	0.0157 (6)	0.0133 (6)	0.0019 (5)	0.0019 (5)	0.0010 (5)
C14	0.0167 (6)	0.0172 (6)	0.0140 (6)	−0.0009 (5)	0.0011 (5)	0.0003 (5)
C15	0.0172 (6)	0.0278 (8)	0.0144 (6)	−0.0019 (6)	−0.0006 (5)	−0.0012 (6)
C16	0.0188 (7)	0.0313 (8)	0.0139 (6)	0.0060 (6)	−0.0011 (6)	0.0017 (6)
C17	0.0229 (7)	0.0205 (7)	0.0177 (7)	0.0063 (6)	0.0027 (6)	0.0049 (5)
C18	0.0160 (6)	0.0162 (6)	0.0182 (6)	0.0009 (5)	0.0014 (5)	0.0010 (5)
C19	0.0147 (6)	0.0162 (6)	0.0141 (6)	0.0033 (5)	−0.0017 (5)	−0.0002 (5)
C20	0.0175 (6)	0.0201 (7)	0.0148 (6)	0.0048 (6)	−0.0009 (5)	−0.0004 (5)
C21	0.0255 (8)	0.0247 (7)	0.0158 (6)	0.0098 (6)	−0.0014 (6)	−0.0045 (5)
C22	0.0323 (9)	0.0201 (7)	0.0248 (7)	0.0053 (7)	−0.0060 (7)	−0.0075 (6)
C23	0.0287 (8)	0.0189 (7)	0.0310 (9)	−0.0037 (6)	−0.0012 (7)	−0.0045 (6)
C24	0.0200 (7)	0.0187 (7)	0.0225 (7)	−0.0004 (6)	0.0026 (6)	−0.0038 (5)
C25	0.0136 (6)	0.0121 (6)	0.0164 (6)	0.0001 (5)	0.0010 (5)	−0.0013 (5)
C26	0.0165 (6)	0.0193 (7)	0.0164 (6)	0.0009 (5)	0.0001 (5)	−0.0032 (5)
C27	0.0202 (7)	0.0238 (7)	0.0201 (7)	0.0013 (6)	0.0057 (6)	−0.0057 (5)
C28	0.0141 (6)	0.0192 (7)	0.0296 (8)	0.0021 (5)	0.0042 (6)	−0.0035 (6)
C29	0.0151 (6)	0.0170 (6)	0.0253 (7)	0.0029 (5)	−0.0039 (6)	−0.0019 (5)
C30	0.0162 (6)	0.0171 (6)	0.0156 (6)	0.0012 (5)	−0.0002 (5)	−0.0010 (5)

### Geometric parameters (Å, º)

**Table:**

O1—C1	1.3086 (18)	C12—H12	0.9500
O1—C3	1.4712 (17)	C13—C14	1.400 (2)
C1—N1	1.2965 (19)	C13—C18	1.4045 (19)
C1—C2	1.482 (2)	C14—C15	1.392 (2)
N1—C6	1.464 (2)	C14—H14	0.9500
N1—C5	1.4683 (19)	C15—C16	1.384 (2)
C2—H2A	0.9800	C15—H15	0.9500
C2—H2B	0.9800	C16—C17	1.385 (2)
C2—H2C	0.9800	C16—H16	0.9500
C3—C4	1.492 (2)	C17—C18	1.389 (2)
C3—H3A	0.9900	C17—H17	0.9500
C3—H3B	0.9900	C18—H18	0.9500
C4—H4A	0.9800	C19—C24	1.402 (2)
C4—H4B	0.9800	C19—C20	1.4028 (19)
C4—H4C	0.9800	C20—C21	1.396 (2)
C5—H5A	0.9800	C20—H20	0.9500
C5—H5B	0.9800	C21—C22	1.383 (3)
C5—H5C	0.9800	C21—H21	0.9500
C6—H6A	0.9800	C22—C23	1.385 (2)
C6—H6B	0.9800	C22—H22	0.9500
C6—H6C	0.9800	C23—C24	1.390 (2)
B1—C25	1.641 (2)	C23—H23	0.9500
B1—C7	1.643 (2)	C24—H24	0.9500
B1—C19	1.647 (2)	C25—C26	1.3963 (19)
B1—C13	1.650 (2)	C25—C30	1.4019 (19)
C7—C8	1.397 (2)	C26—C27	1.392 (2)
C7—C12	1.408 (2)	C26—H26	0.9500
C8—C9	1.394 (2)	C27—C28	1.380 (2)
C8—H8	0.9500	C27—H27	0.9500
C9—C10	1.381 (3)	C28—C29	1.388 (2)
C9—H9	0.9500	C28—H28	0.9500
C10—C11	1.383 (2)	C29—C30	1.386 (2)
C10—H10	0.9500	C29—H29	0.9500
C11—C12	1.388 (2)	C30—H30	0.9500
C11—H11	0.9500		
			
C1—O1—C3	120.57 (12)	C11—C12—C7	122.80 (14)
N1—C1—O1	115.87 (13)	C11—C12—H12	118.6
N1—C1—C2	122.33 (14)	C7—C12—H12	118.6
O1—C1—C2	121.80 (13)	C14—C13—C18	115.19 (13)
C1—N1—C6	122.65 (13)	C14—C13—B1	122.17 (12)
C1—N1—C5	122.11 (13)	C18—C13—B1	122.63 (12)
C6—N1—C5	115.24 (12)	C15—C14—C13	122.79 (14)
C1—C2—H2A	109.5	C15—C14—H14	118.6
C1—C2—H2B	109.5	C13—C14—H14	118.6
H2A—C2—H2B	109.5	C16—C15—C14	120.26 (15)
C1—C2—H2C	109.5	C16—C15—H15	119.9
H2A—C2—H2C	109.5	C14—C15—H15	119.9
H2B—C2—H2C	109.5	C15—C16—C17	118.58 (14)
O1—C3—C4	106.22 (13)	C15—C16—H16	120.7
O1—C3—H3A	110.5	C17—C16—H16	120.7
C4—C3—H3A	110.5	C16—C17—C18	120.59 (14)
O1—C3—H3B	110.5	C16—C17—H17	119.7
C4—C3—H3B	110.5	C18—C17—H17	119.7
H3A—C3—H3B	108.7	C17—C18—C13	122.48 (14)
C3—C4—H4A	109.5	C17—C18—H18	118.8
C3—C4—H4B	109.5	C13—C18—H18	118.8
H4A—C4—H4B	109.5	C24—C19—C20	114.88 (13)
C3—C4—H4C	109.5	C24—C19—B1	119.72 (12)
H4A—C4—H4C	109.5	C20—C19—B1	125.38 (13)
H4B—C4—H4C	109.5	C21—C20—C19	122.59 (14)
N1—C5—H5A	109.5	C21—C20—H20	118.7
N1—C5—H5B	109.5	C19—C20—H20	118.7
H5A—C5—H5B	109.5	C22—C21—C20	120.45 (14)
N1—C5—H5C	109.5	C22—C21—H21	119.8
H5A—C5—H5C	109.5	C20—C21—H21	119.8
H5B—C5—H5C	109.5	C21—C22—C23	118.77 (15)
N1—C6—H6A	109.5	C21—C22—H22	120.6
N1—C6—H6B	109.5	C23—C22—H22	120.6
H6A—C6—H6B	109.5	C22—C23—C24	120.03 (15)
N1—C6—H6C	109.5	C22—C23—H23	120.0
H6A—C6—H6C	109.5	C24—C23—H23	120.0
H6B—C6—H6C	109.5	C23—C24—C19	123.28 (14)
C25—B1—C7	110.73 (11)	C23—C24—H24	118.4
C25—B1—C19	107.60 (11)	C19—C24—H24	118.4
C7—B1—C19	111.72 (11)	C26—C25—C30	115.32 (12)
C25—B1—C13	109.61 (11)	C26—C25—B1	123.41 (12)
C7—B1—C13	108.36 (11)	C30—C25—B1	121.28 (12)
C19—B1—C13	108.78 (11)	C27—C26—C25	122.69 (14)
C8—C7—C12	114.83 (13)	C27—C26—H26	118.7
C8—C7—B1	124.21 (13)	C25—C26—H26	118.7
C12—C7—B1	120.85 (12)	C28—C27—C26	120.27 (14)
C9—C8—C7	122.82 (14)	C28—C27—H27	119.9
C9—C8—H8	118.6	C26—C27—H27	119.9
C7—C8—H8	118.6	C27—C28—C29	118.79 (14)
C10—C9—C8	120.52 (15)	C27—C28—H28	120.6
C10—C9—H9	119.7	C29—C28—H28	120.6
C8—C9—H9	119.7	C30—C29—C28	120.19 (14)
C9—C10—C11	118.46 (14)	C30—C29—H29	119.9
C9—C10—H10	120.8	C28—C29—H29	119.9
C11—C10—H10	120.8	C29—C30—C25	122.72 (13)
C10—C11—C12	120.53 (15)	C29—C30—H30	118.6
C10—C11—H11	119.7	C25—C30—H30	118.6
C12—C11—H11	119.7		
			
C3—O1—C1—N1	176.76 (12)	C16—C17—C18—C13	−0.4 (2)
C3—O1—C1—C2	−4.0 (2)	C14—C13—C18—C17	−2.6 (2)
O1—C1—N1—C6	1.3 (2)	B1—C13—C18—C17	178.54 (13)
C2—C1—N1—C6	−178.02 (14)	C25—B1—C19—C24	−37.11 (17)
O1—C1—N1—C5	−177.86 (13)	C7—B1—C19—C24	−158.86 (13)
C2—C1—N1—C5	2.9 (2)	C13—B1—C19—C24	81.56 (16)
C1—O1—C3—C4	−171.52 (13)	C25—B1—C19—C20	144.72 (13)
C25—B1—C7—C8	−2.65 (18)	C7—B1—C19—C20	22.97 (19)
C19—B1—C7—C8	117.28 (14)	C13—B1—C19—C20	−96.61 (15)
C13—B1—C7—C8	−122.89 (14)	C24—C19—C20—C21	−0.5 (2)
C25—B1—C7—C12	173.30 (12)	B1—C19—C20—C21	177.71 (13)
C19—B1—C7—C12	−66.78 (16)	C19—C20—C21—C22	0.0 (2)
C13—B1—C7—C12	53.05 (16)	C20—C21—C22—C23	0.5 (2)
C12—C7—C8—C9	1.0 (2)	C21—C22—C23—C24	−0.5 (3)
B1—C7—C8—C9	177.16 (13)	C22—C23—C24—C19	0.0 (3)
C7—C8—C9—C10	0.4 (2)	C20—C19—C24—C23	0.5 (2)
C8—C9—C10—C11	−1.5 (2)	B1—C19—C24—C23	−177.81 (14)
C9—C10—C11—C12	1.1 (2)	C7—B1—C25—C26	−113.31 (15)
C10—C11—C12—C7	0.4 (2)	C19—B1—C25—C26	124.32 (14)
C8—C7—C12—C11	−1.4 (2)	C13—B1—C25—C26	6.19 (19)
B1—C7—C12—C11	−177.70 (13)	C7—B1—C25—C30	66.74 (16)
C25—B1—C13—C14	−101.29 (15)	C19—B1—C25—C30	−55.62 (16)
C7—B1—C13—C14	19.65 (17)	C13—B1—C25—C30	−173.76 (12)
C19—B1—C13—C14	141.31 (13)	C30—C25—C26—C27	−0.8 (2)
C25—B1—C13—C18	77.45 (16)	B1—C25—C26—C27	179.30 (14)
C7—B1—C13—C18	−161.60 (12)	C25—C26—C27—C28	−0.2 (2)
C19—B1—C13—C18	−39.95 (17)	C26—C27—C28—C29	0.7 (2)
C18—C13—C14—C15	3.5 (2)	C27—C28—C29—C30	−0.2 (2)
B1—C13—C14—C15	−177.70 (13)	C28—C29—C30—C25	−0.8 (2)
C13—C14—C15—C16	−1.3 (2)	C26—C25—C30—C29	1.3 (2)
C14—C15—C16—C17	−2.0 (2)	B1—C25—C30—C29	−178.78 (13)
C15—C16—C17—C18	2.8 (2)		

### Hydrogen-bond geometry (Å, º)

*Cg*1, *Cg*2 and *Cg*3 are the centroids of the C7–C12, C13–C18 and C25–C30 rings, respectively.

**Table:**

*D*—H···*A*	*D*—H	H···*A*	*D*···*A*	*D*—H···*A*
C3—H3*A*···*Cg*1	0.99	2.67	3.572 (2)	151
C5—H5*B*···*Cg*2	0.98	2.70	3.450 (2)	134
C6—H6*B*···*Cg*3	0.98	2.72	3.692 (2)	175
