# Supplementary figures and images for: Crystal structure of (1-eth­oxy­ethyl­idene)di­methyl­aza­nium tetra­phenyl­borate
Source: Acta Crystallogr E Crystallogr Commun. 2015 Nov 25;71(Pt 12):o984–5. doi: 10.1107/S2056989015022252 (PMC4719936; doi:10.1107/S2056989015022252)

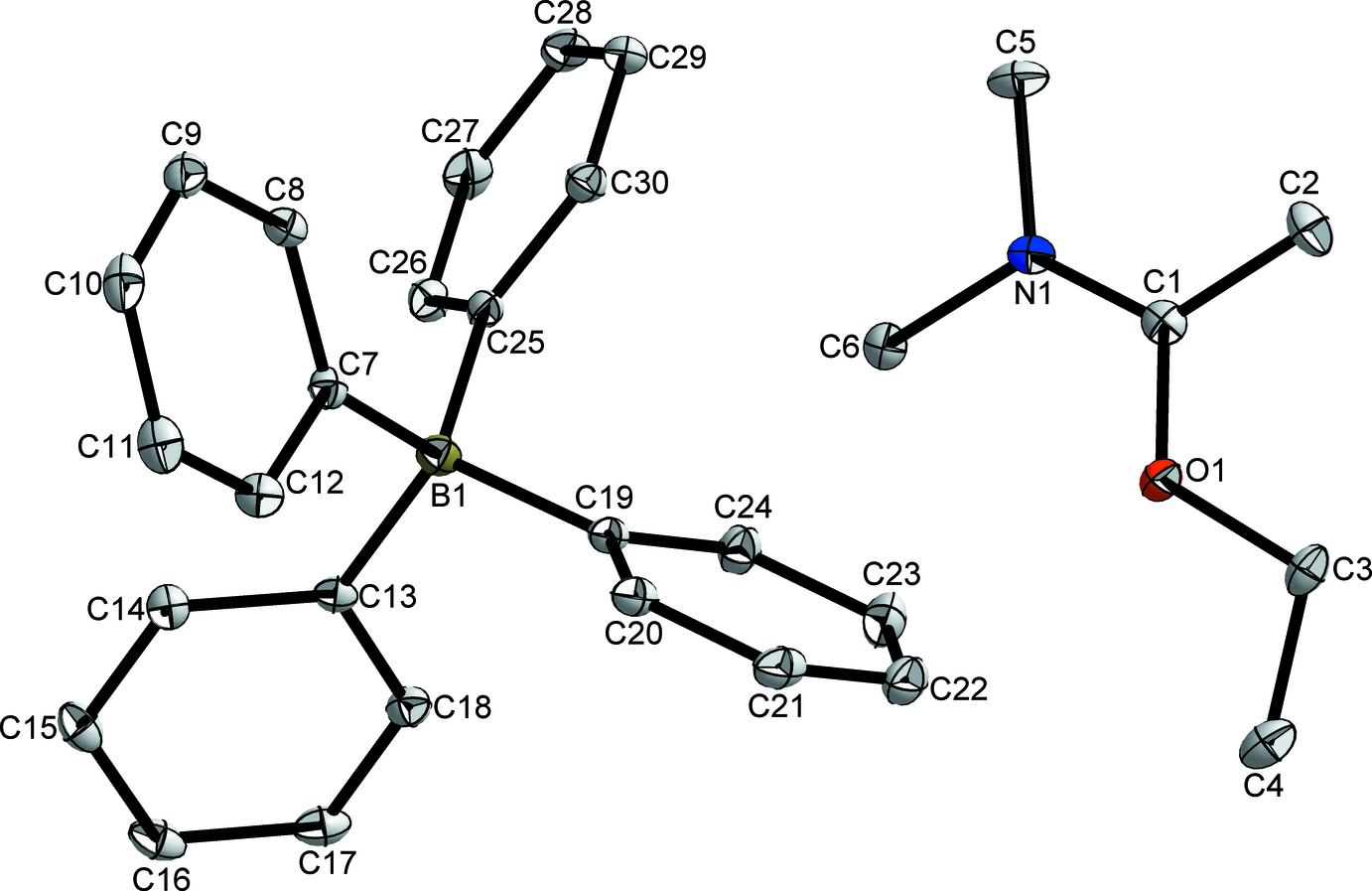

Supplement: Supplementary file 4 [file e-71-0o984-fig1.tif]

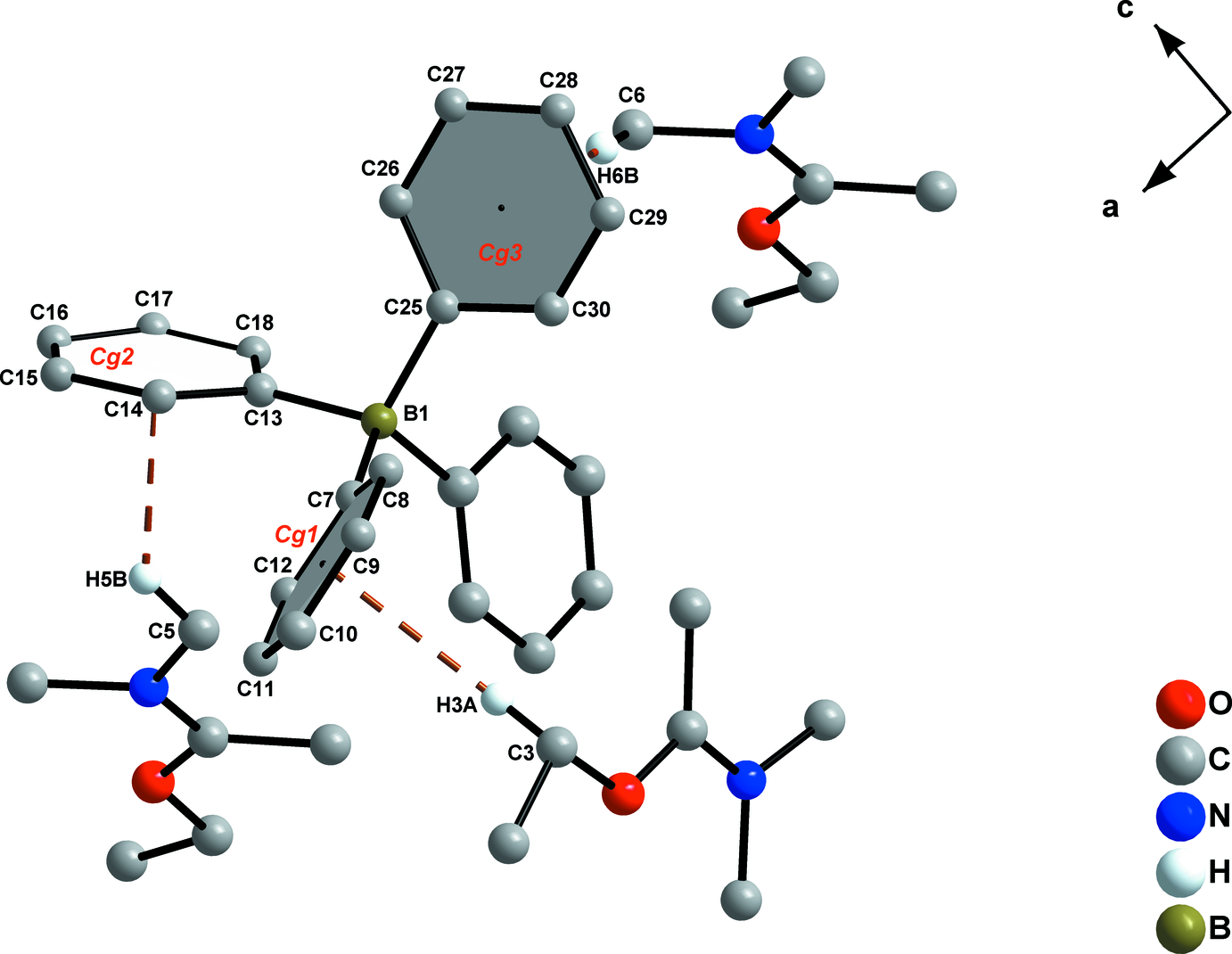

Supplement: Supplementary file 5 [file e-71-0o984-fig2.tif]
